# Anti-Corrosion Performance of Magnesium Potassium Phosphate Cement Coating on Steel Reinforcement: The Effect of Boric Acid

**DOI:** 10.3390/ma17215310

**Published:** 2024-10-31

**Authors:** Fan Zhang, Jihui Qin, Kangyi Cai, John J. Myers, Hongyan Ma

**Affiliations:** 1 Department of Civil, Architectural, and Environmental Engineering, Missouri University of Science and Technology, Rolla, MO 65401, USA; zyjtzf@gmail.com (F.Z.); jqin@mst.edu (J.Q.); kc3kr@mst.edu (K.C.); 2 Henan Zhongyu Construction Investment Group Co., Ltd., Zhengzhou 450001, China

**Keywords:** magnesium potassium phosphate cement, boric acid, plain round reinforcement, coating, anti-corrosion

## Abstract

It has recently been found that magnesium potassium phosphate cement (MKPC) paste coating applied on the surface of steel reinforcement can effectively retard the onset of corrosion and suppress corrosion reactions. However, the fast-setting nature of MKPC—which is a merit in repair—can be problematic in a practical engineering process of coating the steel reinforcement with MKPC paste. To address this problem, boric acid (H_3_BO_3_) was added as a retarder in an MKPC formulation to prolong the setting time. This work investigated the impact of boric acid (at 5% by weight of MgO) on the anti-corrosion performance of MKPC paste coating through a series of electrochemical (EC) tests. The results showed that the anti-corrosion performance of MKPC paste coating for a mild steel bar could be interfered with by the presence of boric acid. In the same testing situation (immersed in 3.5 wt.% NaCl corrosion solution), the polarization resistance and corrosion current density of the group including boric acid were inferior and exceeded the corrosion thresholds prior to the control group without boric acid. Meanwhile, the time constant phase in the frequency range from 1 Hz to 10 kHz was rarely observed, implying that the presence of boric acid probably impaired the formation of the passivation layer. This decrease in anti-corrosion performance of MKPC paste coating could be related to the larger volume fraction of pores in the range from 0.1 to 10 µm that are formed during the initial stage of coating formation.

## 1. Introduction

Magnesium potassium phosphate cement (MKPC) is a well-known repair material in infrastructure engineering due to its fast setting, high early-age strength, high bonding capacity to concrete substrate and steel, low permeability, and superior durability in certain service environments [1,2,3,4]. MKPC is normally proportioned with magnesium oxide calcined at a high temperature of 1500–2000 °C (i.e., hard-burnt or dead burnt magnesia) and acidic water-soluble potassium dihydrogen phosphate, KH_2_PO_4_ (KDP); and its cementation mechanism is an acid–base chemical reaction [5], forming the dominant reaction product of struvite-K (i.e., MgKPO_4_·6H_2_O) as described in Equation (1) [6]:

MgO + KH_2_PO_4_ + 5H_2_O → MgKPO_4_⋅6H_2_O (1)

This reaction is highly exothermic and very fast. When a large volume of materials is produced, the autocatalytic phenomenon can be observed [7]: the chemical reaction releases a large amount of heat to increase the temperature, which further accelerates the acid–base reaction, leading to reaction completion within several minutes. Given the high reactivity of MKPC, a retarder is intensively recommended to mix with MKPC and delay the hydration, so that it can succeed in production and application at scale. Normally, retarders of MKPC are presented in the form of water-soluble fluoride, polyphosphate, or oxy-boron compounds [8]. Common chemicals such as boric acid (H_3_BO_3_), borax (NaB_4_O_7_·10H_2_O), and sodium triphosphate (Na_5_P_3_O_10_) have been employed as retarders in MKPC and widely investigated. Different hypotheses regarding the retardation mechanism of borax and boric acid have been proposed [9]. The most widely accepted theory suggests that these chemicals can be hydrolyzed to yield B_4_O_7_^2–^ ions that react rapidly with initially disassociated Mg^2+^ ions to form compounds that precipitate as a film on magnesia particles to severely delay the reaction between magnesium oxide and phosphate [10,11,12].

In recent years, MKPC paste has been considered as a potential coating of steel rebar to prevent corrosion since it can establish a passive film over the substrate steel surface [13,14]. Several studies have focused on the corrosion resistance performance involved in the passivation process and the formation of the passive layer between the rebar and MKPC paste. It has been found that, for the MKPC with MgO-to-KDP (Mg/P) molar ratios from 7 to 17 [15], the passivation degree remarkedly increased with increasing Mg/P ratio. Iron phosphate and iron hydroxide were detected on the reinforcement surface, which were formed due to the reaction of the steel and MKPC pore solution with a Mg/P ratio of 7. Tang et al. [13,14] reported the same results: the excellent protective ability of MKPC coating was attributed to the formed insoluble iron phosphates and the composite product passivation layer between the reinforcement and MKPC. Moreover, the presence of metakaolin in an MKPC system could improve its pore structure by minimizing the porosity of the coating layer [16], and thus further improve the anti-corrosion capacity of the MKPC coating.

In spite of the high performance of the MKPC anti-corrosion coating observed under lab conditions, where the MKPC paste can be easily produced, coated, and finished, the application of this technology in field conditions could be challenging if the MKPC paste sets too fast. Thus, a retarder, e.g., boron-containing compounds, may be used to prolong the setting time so that the coating process can be readily manipulated. The addition of a retarder would not only modulate the setting time but alter the phase composition and microstructure in the MKPC paste, and therefore may influence its anti-corrosion performance. However, the manner in which the presence of a retarder affects the anti-corrosion capacity of MKPC coating is still unclear. Understanding the influence of a retarder on the anti-corrosion properties of MKPC paste is important for the formulation optimization of MKPC paste with aimed application in the coating field.

The objective of this study is to investigate the effect of boric acid—a widely used and highly effective retarder—on short- and long-term anti-corrosion performance of MKPC paste coating. The coated plain round rebar and its counterparts were immersed in 3.5 wt.% NaCl solution to stimulate corrosion. Electrochemical (EC) tests, including open-circuit potential (OCP), electrochemical impedance spectroscopy (EIS) and polarization resistance (R_P_), were conducted every week for the first month and then every two months up to 10,080 h (420 days) to monitor the evolution of the passivation state and corrosion process. Before EC tests, two types of MKPC coating (with and without retarder) were compared in the light of isothermal calorimetry (IC), X-ray diffraction (XRD), thermogravimetry/differential thermal analysis (TG/DTA), and mercury intrusion porosimetry (MIP) tests. After all the tests, a potentiodynamic polarization (PDP) test and visual inspection were performed to evaluate the degrees of corrosion.

## 2. Experiment Details

### 2.1. Materials

Three major raw materials were used in this study, i.e., dead burned magnesia (DBM), potassium dihydrogen phosphate (KH_2_PO_4_, KDP), and metakaolin (MK). The DBM was provided by Martin Marietta Magnesia Special Ties, LLC (Manistee, MI, USA), and it had a BET specific surface area of 0.742 m^2^/g as measured by a NOVA 600 automated surface area and pore size analyzer (Anton Paar, Austria). The KDP was a chemical regent of over 95% purity, provided by ICL Specialty Fertilizers-Americas (Summerville, SC, USA). The MK had a specific surface area of 12.134 m^2^/g. The chemical compositions of these materials determined using an X-supreme 8000 X-ray fluorescence spectrometer (Oxford Instruments, Abingdon, UK) are shown in Table 1.

Furthermore, reagent boric acid (H_3_BO_3_) powder of more than 99% purity, supplied by Ecoxall Chemicals (Brighton, MI, USA), was used as a retarder in this study. Plain round steel bars with a diameter of 12.7 mm were used as the substrate of the coating as they are widely used in reinforced concrete structures. Meanwhile, deionized (DI) water was used during the entire research process including the preparation of corrosion solution and MKPC paste coating.

### 2.2. Preparation of Coatings and Samples

#### 2.2.1. Preparation of Coating Groups and 3.5 wt.% NaCl Corrosion Solution

This research investigated two testing groups. The first one focused on round steel bars coated with a MKPC paste without a retarder. The paste was prepared with a Mg/P molar ratio of 6, and MK was added at 10% by weight of the total binder. The water-to-binder mass ratio (W/B) used to prepare the paste was 0.2. This group of samples was denoted by ‘MKPC’, serving as the reference group. For the second group of samples, boric acid was added into MKPC paste coating on the basis of the reference proportion. The addition of boric acid resulted in a reduced flowability of fresh MKPC paste. Therefore, 5% boric acid by weight of MgO—instead of a larger dosage—was selected to maintain sufficient workability for coating. This second group of samples was denoted by ‘B-MKPC’.

The 3.5 wt.% NaCl corrosion solution with a chloride concentration of about 0.6 mol/L was made by mixing pure sodium chloride with DI water. After magnetic stirring in an indoor environment [temperature of 20 ± 2 °C and relative humidity of 55 ± 10%], this solution was used to soak samples for corrosion tests. Before EC tests, the electrical conductivity and temperature of 3.5 wt.% NaCl corrosion solution were determined by an HI5522 Meter (Hanna Instruments, Woonsocket, RI, USA). These two values stabilized at 55.0 ± 5.0 mS/cm and 20 ± 2 °C, respectively. During the entire test period, the conductivity and temperature of the corrosion solution were measured in the same way. It was found that these two values remained constant in the testing period. Furthermore, the pH value of the corrosion solution with coated samples was also monitored, and it was kept in the range of 8.5–9.0. These corrosion solutions were contained in beakers, where the coated rebars were immersed. These solutions were directly exposed to the indoor environment, along with the immersed coated sample. Therefore, one should expect that the oxygen and carbon dioxide in the air could be dissolved into the solution and took part in the steel corrosion process [17,18].

#### 2.2.2. Preparation of Electrochemical Specimens

**Figure 1:**
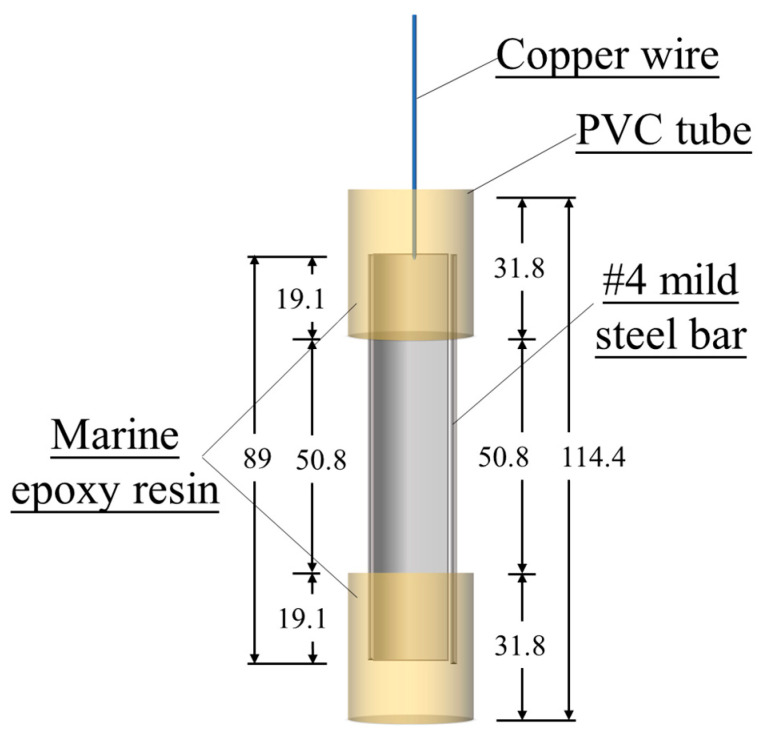
Geometry of rebar samples (unit: mm).

The diagram of the electrochemical specimen is shown in Figure 1. The two ends of the plain round rebar with a length of 89 mm were encased in PVC tubes that were filled with marine epoxy resin to physically isolate and prevent electric conductivity with the outside aggressive corrosion environment. Each of the PVC tubes was 31.8 mm in both height and diameter, and the actual coating length of sample steel bar was 50.8 mm in the middle portion, which had a testing surface area of approximately 20.26 cm^2^. A copper wire was electrically connected at one end of the mild steel bar.

All the mild steel bars used to make specimens were cut from same rebar and treated by using sandpaper before the coating process, which included sequential polishing from 240 to 1000 grit to remove the oxide layer and small rust spots. Subsequently, they were cleaned with pure-100 isopropyl alcohol solution in an ultrasonic cleaner for 60 s, and then dried in a vacuum drying oven at 100 °C for 4 h. The two groups of samples were prepared as follows: the paste layer with a thickness of 1.0 ± 0.3 mm was coated on the testing portion of the treated rebar and exposed to the indoor environment for 7 days before samples were immersed into the corrosion solution for stimulating corrosion and conducting EC tests.

### 2.3. Test Method

#### 2.3.1. Characterization of Coating Layer

The setting time of MKPC paste samples was determined by the Vicat needle apparatus in accordance with ASTM C191 [19]. Considering that the time interval between the initial and final setting was too short, only the initial setting time was recorded to represent its setting and hardening characteristic. The setting time was around 2.7 min for the MKPC group and 12.5 min for B-MKPC. At the age of 7 days, cubic specimens with dimensions of 20 × 20 × 20 mm were used to measure compressive strength. The strengths of MKPC and B-MKPC groups were 36.8 and 36.3 MPa, respectively, showing that the use of boric acid as a retarder does not have an obviously negative effect on 7-day compressive strength.

The isothermal calorimetry (IC) test was carried out using an I-Cal 8000 isothermal calorimeter (Calmetrix, Arlington, MA, USA) to monitor the initial hydration rate of MKPC paste through quantifying the thermal production rate, which is compliant with ASTM C1679 [20]. Before the test, the materials, container, and stirring rod were placed into the calorimetry chamber for several hours to let them reach temperature equilibrium. Once the temperature stabilized, the prepared materials were rapidly mixed with water and then placed into the testing cell for 24 h.

In order to determine the phase composition and porosity, the coating layer of each sample after 7 days of curing was peeled from the rebars and immersed in pure-100 isopropyl alcohol solution for 48 h to replace the water and stop hydration. Then, the coating samples were vacuum dried at 30 °C for 48 h. Before X-ray diffraction (XRD) and thermogravimetry/differential thermal analysis (TG/DTA) tests, the treated samples were further ground to pass through a 75 µm sieve. The XRD data were collected by a PANanalytical X’pert Pro MPD diffractometer (Malvern Panalytical, Almelo, The Netherlands), using CuKα radiation (λ = 1.54 Å). The patterns were examined with 2θ ranging from 5° to 90° for 15 min. The phase compositions were identified with the assistance of X’pert high score Plus software (version 3.0.5). TG/DTA tests were conducted on the prepared powders by using an SDT Q600 thermogravimetric analyzer (TA instruments, New Castle, DE, USA) under nitrogen atmosphere with a flowing rate of 40 mL/s. These powders were heated from room temperature to 30 °C with a duration of 5 min, and then heated to 850 °C at a heating rate of 10 °C/min in an aluminum crucible. The porosity of the treated samples was measured with the mercury intrusion porosimetry (MIP) test by a PoreMaster 60 porosimeter (Quantachrome Instruments, Boynton Beach, FL, USA). A maximum pressure of 206.8 MPa was applied in the MIP test. The total porosity volume was determined from the total mercury volume intruded and the volume intruded at each pressure increment. The accumulation of porosity curves was displayed and analyzed.

#### 2.3.2. Electrochemical Measurements

**Figure 2:**
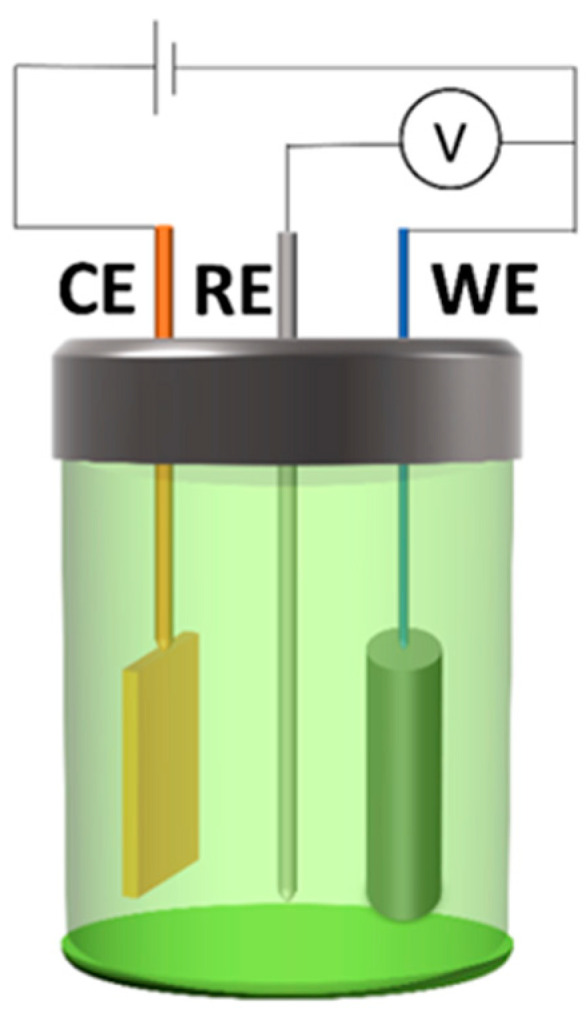
Three-electrode setup.

The testing data from the EC test displayed the anti-corrosion performance of the MKPC paste coating, which was measured and collected by using a typical three-electrode setup named SP-105 Potentiostat (BioLogic, Seyssinet-Pariset, France). In this work, the three-electrode system included a 25.4 × 25.4 × 0.254 mm platinum sheet as the counter electrode (CE), a saturated calomel electrode (SCE) as the reference electrode (RE) and the coated prepared specimens as the working electrode (WE). The spacing length between all three electrodes was around 20 mm. A schematic diagram of the EC test setup is shown in Figure 2. After 7 days of curing, the coated samples were immersed in 3.5 wt.% NaCl solution consisting of distilled water and purified sodium chloride. Electrochemical impedance spectrometer (EIS) data began at 168 h (7 days), and all other electrochemical data collected in this paper were recorded for increasing immersion times at 5 h and then at 168 h (7 days) up to 672 h (28 days), followed by 1344 h (56 days) up to 10,080 h (420 days). Before and during EIS spectra recording, the coating layers soaked for different periods of time were fully water saturated. For each group, three specimens were tested to record the variability of test data. Since the data from the same group in the same situation exhibited a similar trend, only the representative results of each group have been presented in this paper.

The electrochemical behaviors of coated plain round rebar samples during the entire testing process were monitored using electrochemical test methods, i.e., open-circuit potential (E_ocp_), polarization resistance (R_P_) [21], and electrochemical impedance spectrometer (EIS) [22]. E_ocp_ data were recorded every 1 s. The first-time testing lasted for 3600 s, and the remaining tests continued until the |dE_we_/dt| (mV/h) was less than 5. As the immersion time increased, the E_OCP_ value tended to stabilize. R_P_ data were calculated according to the inversion of the slope of the current–potential curve. The curve was recorded within E_ocp_ ± 15 mV at a scan rate of 0.167 mV/s, and its corresponding potentials were set with respect to the measured E_ocp_. EIS was tested with an applied sinusoidal potential wave of 10 mV amplitude and a frequency range of 100 kHz to 0.01 Hz at a sampling of 6 data cycles/decade after the polarization resistance test. EC-lab software (version 10.37) was used to fit the collected EIS data using the electrical equivalent circuit (EEC) model.

#### 2.3.3. Further Analyzing the Corrosion Rate and Degree

The data extracted from the measured potentiodynamic polarization (PDP) [23] plots were used to analyze the corrosion degree and rate. The same samples used in the previous EC test were tested with the PDP from E_ocp_−250 mV to E_ocp_+700 mV with a scanning rate of 5 mV/s at 840 h (35 days), 5376 h (224 days), and 10,080 h (420 days). Furthermore, the corrosion morphology and the steel surface between the two group samples, MKPC and B-MKPC, and testing situation are shown in this research to compare the corrosion degree between the two groups. In addition, the inner sides of coating layers immersed in a corrosion solution for 10,080 h (420 days) were examined by an RH-2000 3D digital microscope (Hirox, Oradell, NJ, USA) with a magnification of 500 and 2000 times.

## 3. Results and Discussions

### 3.1. Microstructure of Coating Layer

The protection ability of coatings is directly related to the coating microstructure (i.e., the hydration process, porosity and chemical composition). In order to more precisely evaluate and better understand the corrosion resistance of two groups of coatings, the characterizations of each sample were performed by studying their early hydration behaviors, phase composition and porosity.

#### 3.1.1. Early Age Hydration Behavior

**Figure 3:**
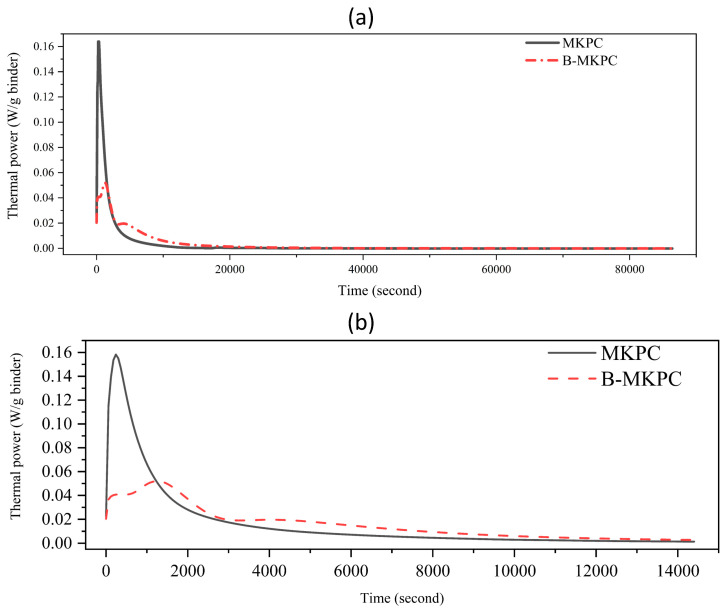
Hydration heat flow of MKPC and B-MKPC (**a**): 86,400 s and (**b**): 14,400 s.

Figure 3a illustrates the hydration flow of the MKPC and B-MKPC samples within 24 h and the data for the first 4 h heat flow is expended in Figure 3b. For the MKPC sample, the hydration heat releasing rate increased sharply at the first hundreds of seconds, with a single exothermic peak achieved around 300 s due to the rapid acid–base reaction. When the boric acid was introduced into the reference system, the initial exotherm was separated into two peaks, with the main peak located between 1000 and 2000 s and the second one at around 4200 s. In Figure 3b, there was a shoulder (about 300 s) to the main peak, which was likely associated with the initial MgO dissolution, prior to the formation of struvite-K at 1400 s. A second exothermic peak was observed at 4200 s, which likely corresponded to the continued struvite-K formation from the MgO reaction with phosphate in the pore solution [24]. This hydration process is in good agreement with the previous research [7], which concluded that boric acid retards the formation of the hydration products. Therefore, the presence of boric acid can delay the setting time, implying that mass production is feasible in the working field. On the other hand, the addition of boric acid can limit the heat release in a short time. But the heat-releasing benefit of a coating is not as useful as larger volume engineering, since the thickness of the MKPC coating layer is just around 1 mm.

#### 3.1.2. Reaction Products

**Figure 4:**
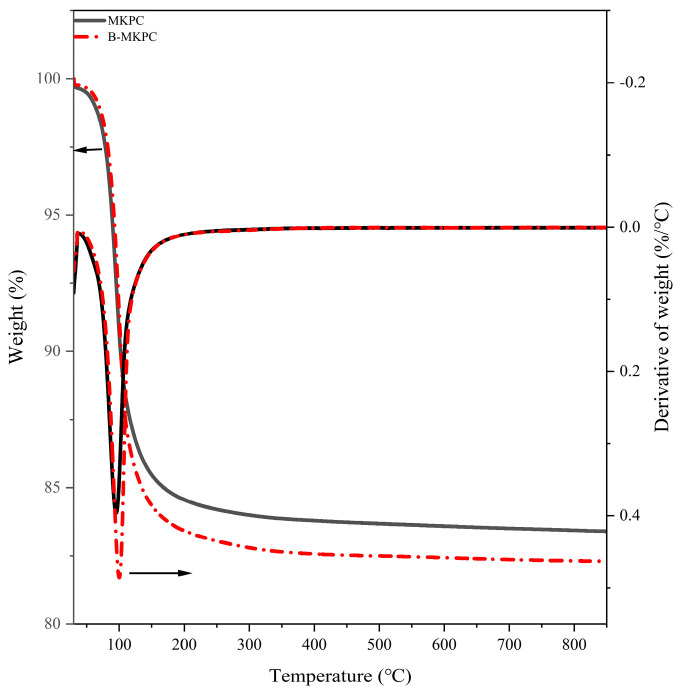
TG/DTA of MKPC (black lines) and B-MKPC (red lines) samples cured for 7 days.

Figure 4 shows the TG/DTA plots of the MKPC and B-MKPC samples cured for 7 days. For both samples, a single weight loss event appeared before 200 °C, which mainly refers to the dehydration of struvite-K. The presence of boric acid in MKPC system did not create a new weight loss peak, indicating that adding boric acid has little influence on the hydration products. However, the B-MKPC sample had a slightly higher weight loss peak than the MKPC sample, indicating that the former produced more products than the latter. This might be due to the longer hydration process time of the B-MKPC sample than the MKPC sample at the stage of saturation and crystallization of the gel, as evidenced in Figure 3.

**Figure 5:**
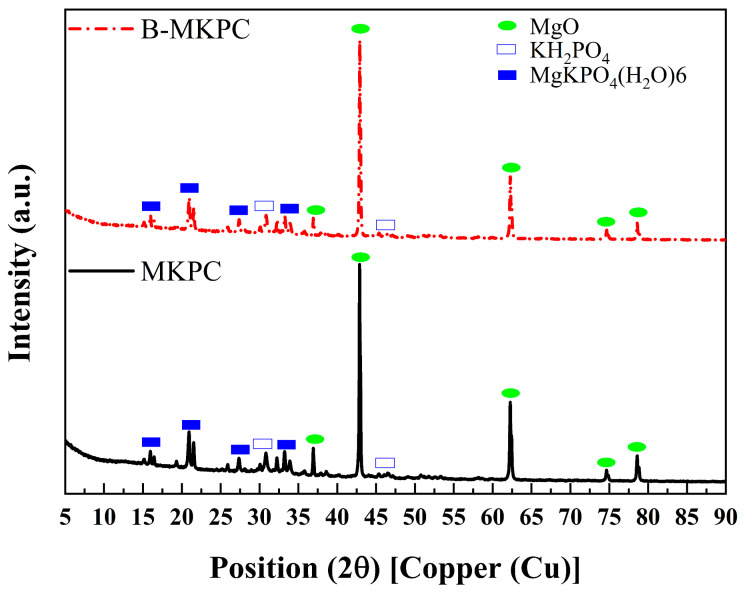
XRD patterns of MKPC and B-MKPC samples after 7 days of curing.

The XRD patterns of the MKPC and B-MKPC pastes after 7 days of curing are presented in Figure 5. The diffraction peaks appearing between 40° and 70° 2θ in the patterns refer to unreacted MgO (PDF #45-0946). Meanwhile, unreacted KDP (PDF #35-0807) can be detected in both samples, likely due to the relatively short curing time. Moreover, the dominant crystalline phase, struvite-K (MgKPO_4_·6H_2_O, PDF #35-0812), was very similar for two samples. Compared with the two diffraction patterns, there were no new crystalline phases resembling some new hydration products after adding boric acid, indicating that the B-MKPC paste coating still has the same chemical composition as the reference one.

According to the previous analysis, the presence of boric acid in the MKPC sample yielded more hydration products. It is common sense that greater hydration production means a denser microstructure of the materials, in addition to improved anti-corrosion performance. However, the following analysis produces the opposite result; mixing boric acid into the MKPC system can negatively affect the porosity, further deteriorating the corrosion resistance ability.

#### 3.1.3. Porosity

**Figure 6:**
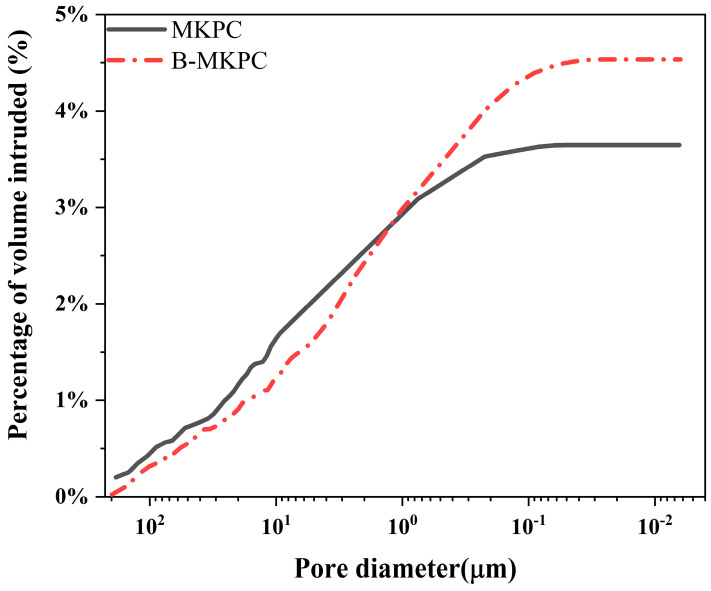
MIP results expressed by cumulative porosity curves for MKPC and B-MKPC samples after 7 days of curing.

Figure 6 presents the cumulative porosity curves of MKPC and B-MKPC samples at the curing age of 7 days. For the MKPC sample, the total porosity was 3.6%, with an extremely low volume of pores with diameters less than 0.1 µm. In comparison to the MKPC, the B-MKPC samples showed a slightly lower volume of pores with diameters less than 10 µm, but a significantly higher volume of pores with diameters less than 1 µm. Finally, the cumulative porosity of B-MKPC reached 4.5%. The formation of these pores is likely to be due to the dissolution of Mg_3_ (PO_4_)·B_2_O_3_·8H_2_O and this process has not been thoroughly investigated yet [9]. The relative higher total porosity of the B-MKPC sample influencing its protection ability was evidenced by the changes in coating layer resistance, as explained in the following sections.

### 3.2. Corrosion Resistance Performance

#### 3.2.1. Open-Circuit Potential

**Figure 7:**
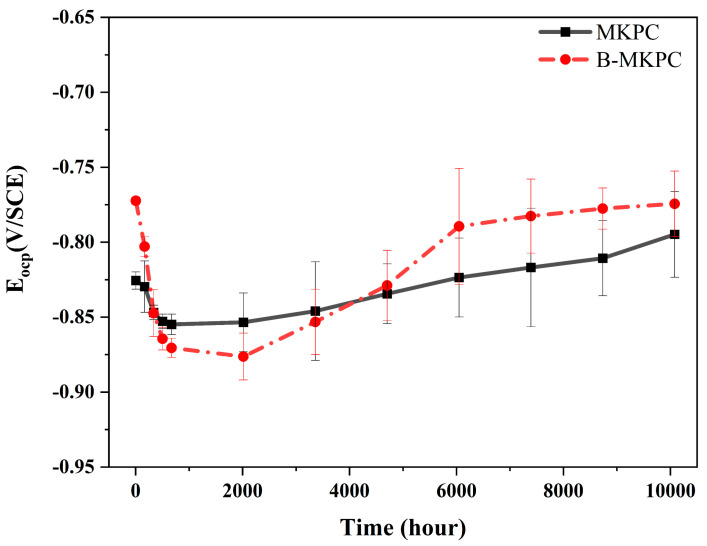
Evolution of open-circuit potential for 10,080 h (420 days).

Figure 7 shows the evolution and standard deviation of open-circuit potentials (E_ocp_) for plain round reinforcement coated with MKPC and B-MKPC in 3.5 wt.% corrosion solution. Two groups of samples presented similar changing trends during the entire testing period. For the MKPC samples, the initial E_ocp_ value was around −0.825 V/SCE after immersing samples for 5 h, which was slightly lower than that of B-MKPC −0.775 V/SCE). Moreover, the E_ocp_ values of these two samples further dropped to the range of −0.85 to −0.875 V/SCE within 672 h (28 days). After that, the E_ocp_ values shifted to less negative values. At the time of 4704 h (196 days), the E_ocp_ value of B-MKPC exceeded that of MKPC, implying that B-MKPC has a lower probability of active corrosion than the reference sample. The two groups of samples tended to stabilize in a low range of −0.775 to −0.8 V/SCE, indicating that a chemical reaction occurred. According to ASTM C876 [25], if potentials over an area are more negative than −0.2735 V/SCE (−0.35 V/CSE), there is a greater than 90% probability that reinforcing steel corrosion is occurring, which means the charge transfer happened between the steel surface and electrolyte or coating layer. Choundhary et al. [26] reported that the E_ocp_ value of uncoated bare steel is higher than −0.7 V/SCE. It is believed that the E_ocp_ values of two groups coating were affected by not only the interaction between the steel and electrolyte, but also the reaction process of the coating layer with iron. Therefore, a drop in the absolute value of E_ocp_ is likely related to the accumulation of the iron compounds.

#### 3.2.2. Electrochemical Impedance Spectroscopy (EIS) Results

**Figure 8:**
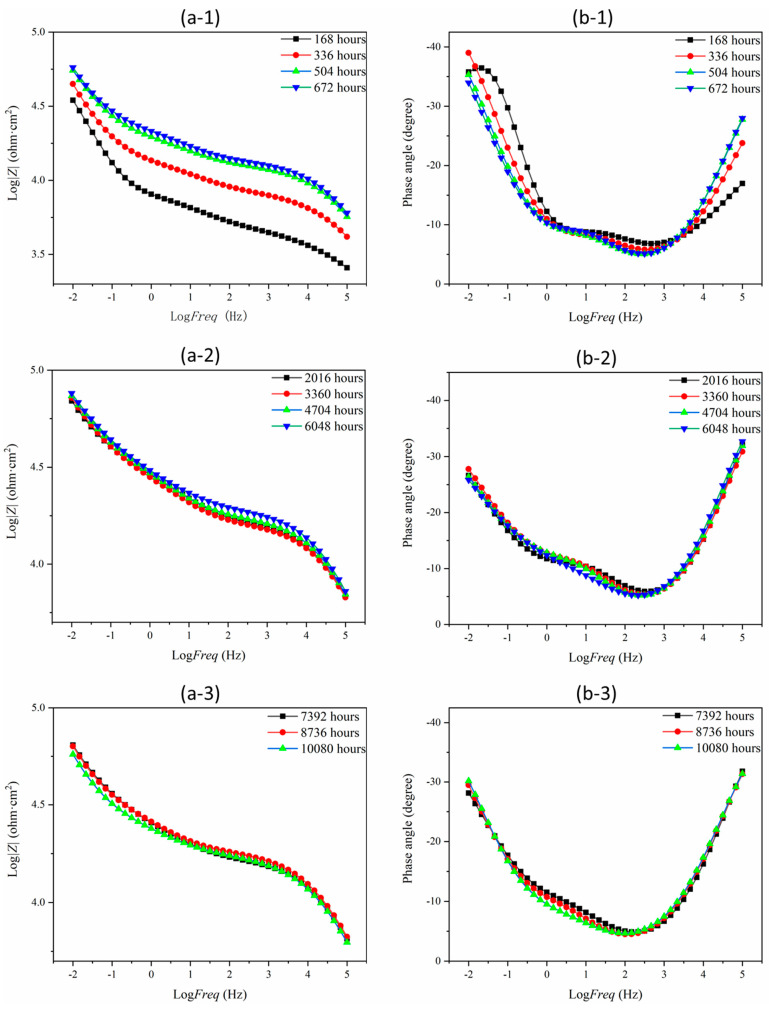

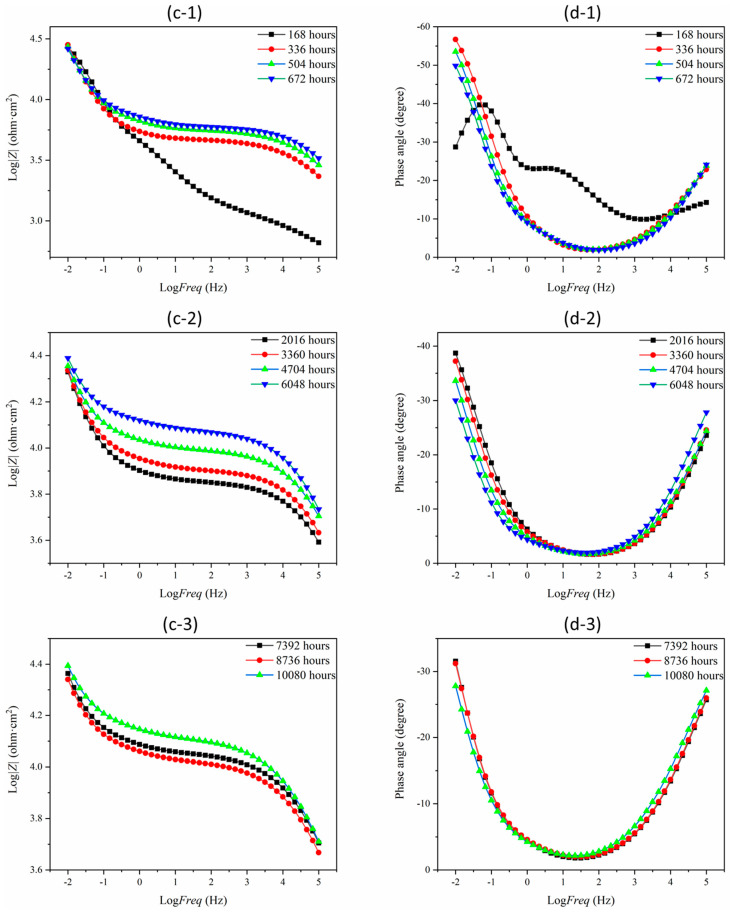
Fitted EIS diagrams (number 1: testing time at 168, 336, 504 and 672 h; number 2: testing time at 2016, 3360, 4704 and 6048 h; number 3: testing time at 7392, 8736 and 10,080 h) for (**a**,**b**) the Bode plots of MKPC sample and for (**c**,**d**) the Bode plots of B-MKPC sample.

**Figure 9:**
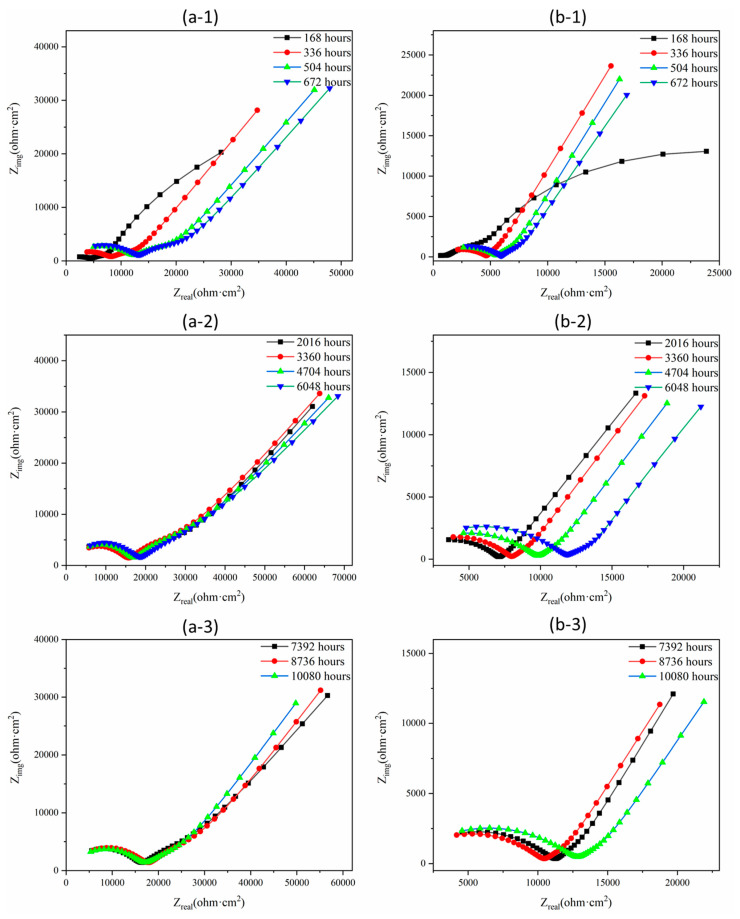
Fitted EIS diagrams (number 1: testing time at 168, 336, 504 and 672 h; number 2: testing time at 2016, 3360, 4704 and 6048 h; number 3: testing time at 7392, 8736 and 10,080 h) for (**a**,**b**) the Nyquist plots of MKPC and B-MKPC sample.

**Figure 10:**
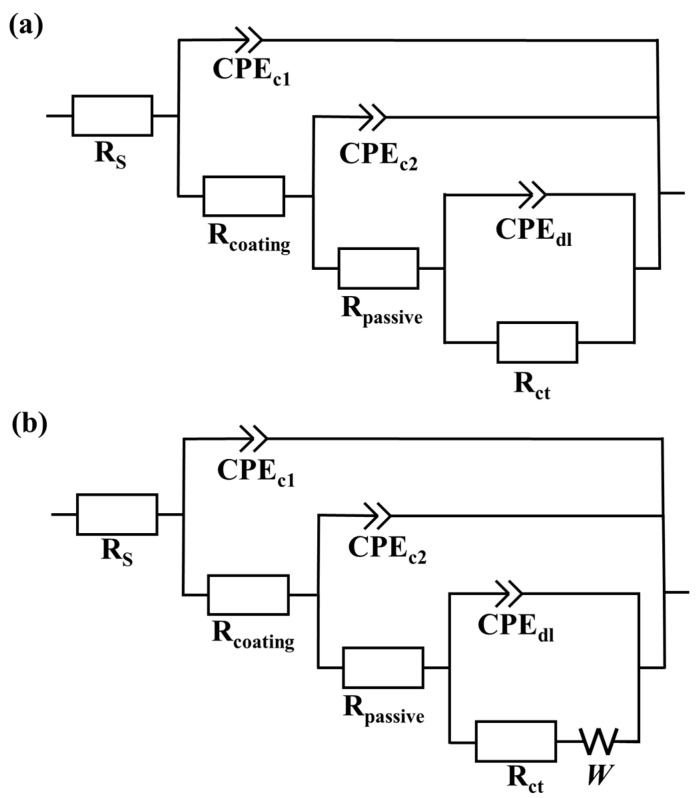
Equivalent electrical circuit (EEC) model: (**a**) EEC model without Warburg element and (**b**) EEC model with Warburg element.

The corrosion process of the representative MKPC and B-MKPC samples immersed in 3.5 wt.% NaCl solution for up to 10,080 h (420 days) was characterized by EIS, and the results presented in the format of both Bode and Nyquist plots are shown in Figure 8 and Figure 9, respectively. Each fitted plot consists of three parts: (a) the fitted measured spectra of every 168 h (7 days) from the immersed 168 h (7 days) to 672 h (28 days), (b) four sets of data from 2016 h (84 days) to 6048 h (252 days) every two months; (c) the spectra of every two months from 7392 h (308 days) to 10,080 h (420 days). Two EEC models were proposed in this study to fit the MKPC and B-MKPC spectra, as summarized in Figure 10.

As indicated in the Bode spectra of Figure 8b, the MKPC sample showed three humps. With the frequency increasing from the left to the right across the X-axis, three time constants can be easily observed from the fitted spectra. The first time constant in the range of high frequency, which is higher than 10 kHz, is associated with the properties of MKPC paste coating. Its corresponding equivalent electrical circuits (EECs) are CPE_c1_ and R_c1,_ as shown in Figure 10, which represent the capacitive and resistive behavior of the coating layer, respectively. The following time constant in the range from 1 Hz to 10 kHz originates from the dielectric properties of the passive layer between the substrate steel and the MKPC paste coating. The related EECs are CPE_c2_ and R_c2,_ as shown in Figure 10, which are used to express the dielectric properties of the passive film. The last time constant, with a frequency lower than 1 Hz, is closely related to the interface properties between the steel and electrolyte or coating layer where corrosion occurs. The corresponding EECs are the CPE_dl_ and R_ct_, which represent the double layer capacitive behavior and charger transfer resistance, respectively. Similar EEC models with three time constants have been used in a previous study on ordinary Portland cement coating and enamel coating corrosion [27,28]. The surface of the working electrode in this study was not a flat plane with complex and small disordered cracks. Similar to the surface of the working electrode, the surface of substrate steel where the chemical reaction happens, and the inner coating surface were also not flat planes. Thus, the constant phase element (CPE), which is normally used to simulate non-homogeneity double-layer capacitance, was employed to illustrate dielectric properties between the two different surfaces. Equation (2) can be used to mathematically calculate the result of the constant phase element (*Z*_CPE_) [29]:

*Z*_CPE_ = *Y*^−1^ ⋅ (*jω*)^−*n*^ (2)

where *Y* (unit is Ω^−1^·cm^−2^·s*^n^*) is a constant phase element and its capacitor is in proportion to the double-layer capacitance of a perfect capacitive electrode, *n* is the control quantity ranging from 0 to 1, $j=\sqrt{-1}$ is the imaginary unit, and the angular frequency of the applied alternative current (AC) is represented by *ω* (*ω* = 2π*f*, the unit of *f*: Hz). Meanwhile, the solution resistance is a significant factor in this three-electrode test. Therefore, it is necessary to consider the electrolyte resistance in the process of building the EEC model. According to Wu [30], the electrolytic resistance (*R*_x_) can be expressed by Equation (3):

*R*_x_ = *J*⁄*κ* (3)

In this equation, κ (S/cm) is the electrolytic conductivity and *J* (cm^−1^) is the constant of the cell, which was calculated based on *J* = *D*/*A*. *D* (cm) is the length between the working electrode and reference electrode and *A* (cm^2^) is the effective area of the electrode. However, the exact calculated value cannot be achieved in practice because of the difficulty in determining the current flow path and the geometry of the electrolyte that carries the current and other factors such as the changes in the temperature and humidity during testing. Therefore, *R*_s_, the electrolyte resistance, was determined when fitting an EEC model to experimental EIS data. The *R*_s_ in the EEC model is shown in Figure 10.

In order to illustrate the goodness of fitted measured data, the *χ*^2^/|*Ƶ*| values for fitting EEC models in the corresponding Nyquist plots are not higher than 10^−3^, which can be expressed by Equation (4):

(4) χ2|Ƶ|=∑i=1n|Ƶmeasi−Ƶsimul(fi,param)|2|Ƶmeas(i)|

The W in the EEC model is the Warburg element, which was used to simulate semi-infinite linear diffusion. It was employed due to the formation and accumulation of some chemical reaction products on the surface of substrate steel. However, for the B-MKPC samples, it is noted that the second time constant corresponding to the passive film layer tended to weaken after 168 h, as shown in Figure 8(d-1). Meanwhile, according to Figure 9(a-1,b-1), the diffusion process did not appear in the first-time testing results of both MKPC and B-MKPC samples, since the slope of Nyquist plots did not align with the diffusion process. Therefore, the EEC model (I) without a Warburg element was used to fit the spectrums of MKPC and B-MKPC samples at 168 h. The weakening film is ascribed to the continuous penetration of chloride and the reaction between MKPC paste and substrate steel. Even the second constant phase tended to be weak and the impedance in the medium frequency range of the Bode plots shown in Figure 8c did not present the two time constant properties. Combined with the fitting results, the time constant related to the passive film retained the same dielectric properties. Thus, the EEC model (II) with three pairs of CPEs and corresponding impedance plus the electrolyte resistance and the Warburg element was used to fit the remained Nyquist spectrum until 10,080 h (420 days).

As shown in Figure 8a for the MKPC sample, a gradual increase in impedance at high frequencies could be observed within first 672 h (28 days), which is related to the further hydration of raw materials. Meanwhile, the impedance value remained stable until the end of the test. For the B-MKPC sample shown in Figure 8c, the impedance at the same frequency dramatically increased during the first 672 h (28 days), and sightly increased in the following tests. The presence of boric acid in MKPC paste coating influences its long-term corrosion resistance performance. Moreover, the impedance at the three frequency bands tended to stabilize until the end of the test for the MKPC sample. However, as indicated in Figure 8b, the hump of the second time constant gradually reduced with a slight shift in the peak, which is probably attributed to the accumulation of iron compounds. For the Nyquist plots shown in Figure 9a,b, the small semi-arc is related to the dielectric properties of the coating layer, where the larger one is associated with the dielectric properties of the passive film and the double layer properties, the changing trend is consistent with the trend from the Bode spectrums.

**Figure 11:**
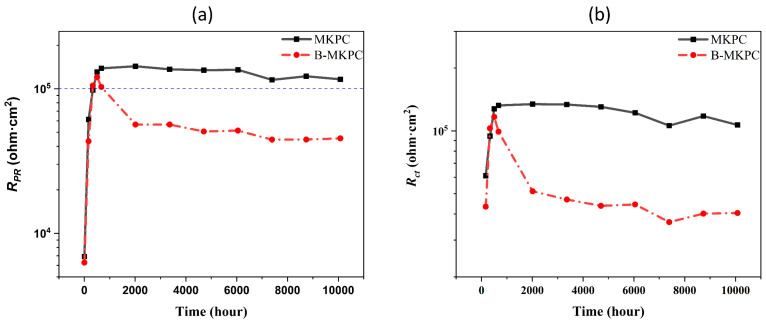
Evolution of (**a**) *R*_p_ (from 5 h) and (**b**) *R*_ct_ (from 168 h) values of MKPC and B-MKPC samples.

**Figure 12:**
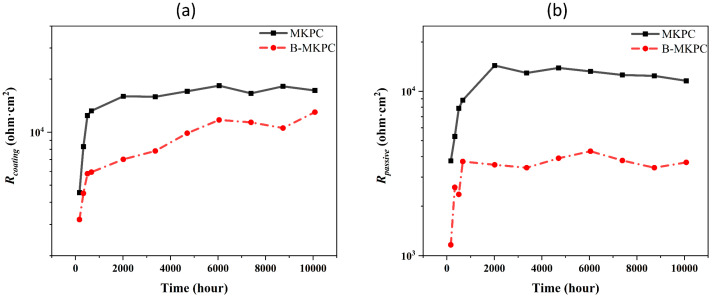
Evolution of (**a**) R_coating_ and (**b**) R_passive_ values of MKPC and B-MKPC samples from 168 h.

To further quantify the corrosion resistance of two types of coatings, the measured polarization resistance (*R*_p_) value shown in Figure 11 was compared with the fitted *R*_ct_ value. A higher *R*_p_ value of the testing material implies higher corrosion. Meanwhile, Figure 12 shows the corresponding values of *R*_passive_ and *R*_coating_ for each sample, which were fitted according to the above EEC models. Normally, the R_ct_ also reflects the ease of charge across the interface where the corrosion occurs [31], and the *R*_p_ value is equal to the *R*_ct_ only if the diffusion does not affect the testing system [32]. Based on the previous section, the *R*_ct_ was lower than the measured R_p_ due to the accumulation of the iron compounds. This aligns well with the results of a previous study [13]. To evaluate the transition of steel corrosion from the passive to active state, the threshold values of *R*_p_ [33], which is 0.1–1 Mohm·cm^2^, was marked on Figure 11a by using a blue dash line. The initial *R*_p_ values of MKPC and B-MKPC were around 6000 ohm·cm^2^ when the samples were immersed into corrosion solution for 5 h, and both samples passed the threshold at the time of 336 h. However, for the B-MKPC sample, a significant drop in the R_p_ value at the time from 672 h to 2016 h was observed and combined with impedance evolution at the low frequency, as shown in Figure 8c. This inconsistent behavior means even though the impedance seems to stabilize during the testing process, R_p_ gradually decreases after 672 h. Therefore, the gap between the real R_p_ and the impedance shown in the Bode spectrum was covered by the increase in dielectric properties of the coating. This was confirmed by the above analysis. On the other hand, for the sample of MKPC, after an increase in the first month, the R_p_ value slightly fluctuated until the end of the test, which means that the system is stable and the substrate steel is in the passive state. Figure 12 shows the evolution of *R*_coating_ and *R*_passive_ of two groups of coating, which could represent a degree of resistance to the penetration of electrolyte and aggressive ions. For two samples’ *R*_passive_ value, the rise during the first month is significant, becoming stable after the first month. Similar changes can be observed for the *R*_coating_ value of MKPC, implying that the passive state was effective. However, as discussed above, for the sample blended with boric acid, even though the R_coating_ maintains stable growth until the end of the test, the corrosion resistance of the B-MKPC coating system shows the opposite result.

In summary, the results show that both the MKPC and B-MKPC samples have good anti-corrosion resistance. However, compared with all the EIS and polarization resistance data, the MKPC coating sample is more favorable for corrosion protection than the B-MKPC coating sample.

### 3.3. Analysis of the Corrosion Rate and Degree

#### 3.3.1. Potentiodynamic Polarization

**Figure 13:**
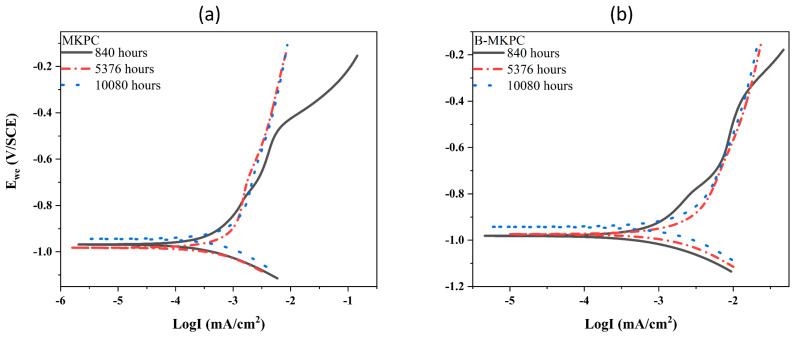
Potentiodynamic polarization curves for (**a**) MKPC samples and (**b**) B-MKPC samples at 840, 5376 and 10,080 h.

Corrosion is a normal, naturally occurring electrochemical reaction between a material, usually a metal/alloy, and its environment, resulting in the deterioration of the metal and its properties. Polarization resistance and potentiodynamic polarization (PDP) tests were carried out to evaluate the corrosion degree and rate of bars under the protection of the MKPC coating. The polarization resistance was discussed in the previous section. Figure 13 shows the PDP curves of MKPC and B-MKPC samples at three testing ages (840, 5376 and 10,080 h). Table 2 presents the values of corrosion current density (*I*_corr_) and corrosion potential (*E*_corr_) that were extracted from PDP plots, and the corrosion rate (CR) was calculated based on Equation (5), as shown below:

(5) $$CR=\frac{I_{c}\cdot K\cdot EW}{d\cdot A}$$

where *CR* is corrosion rate, *I*_c_ is the corrosion current in amperes, *K* is the constant that defines the units of the corrosion rate, *EW* is the equivalent weight (in g/equivalent), *d* is the density, and *A* is the sample area.

The threshold of *I*_corr_, 0.5 µA·cm^−2^ [34], was used to evaluate the corrosion state of the sample. The *I*_corr_ of the MKPC sample was lower than the thresholds at the time of 840 and 5376 h, which were 0.42 and 0.48 µA/cm^2^, respectively. This indicates that the samples remained in the passive corrosion state. With the progress of corrosion, the current density was higher than the threshold of the final time test. The samples with boric acid had an *I*_corr_ that was larger than the threshold from 0.85 µA/cm^2^ at 840 h to 1.49 µA/cm^2^ at the end of the test (10,080 h), implying an active corrosion state. The corrosion rate corresponds to the changing trend of current density; thus, the same results were obtained. As mentioned previously, the chemical composition and coating thickness for the two samples were the same, while the B-MKPC showed larger hydration production but lower total porosity than the MKPC. This implies that the porosity is likely to influence the anti-corrosion performance. In summary, the relative lower corrosion resistance of the B-MKPC paste coating sample can be attributed to the lower hydration rate and higher porosity of the coating layer at the initial stage, which leads to lower total impedance, which includes coating resistance, passive film resistance and charge transfer resistance, of B-MKPC than that of the MKPC. Thus, the B-MKPC had worse anti-corrosion performance than MKPC sample without boric acid since the beginning of the EC test.

#### 3.3.2. Visual Inspection

**Figure 14:**
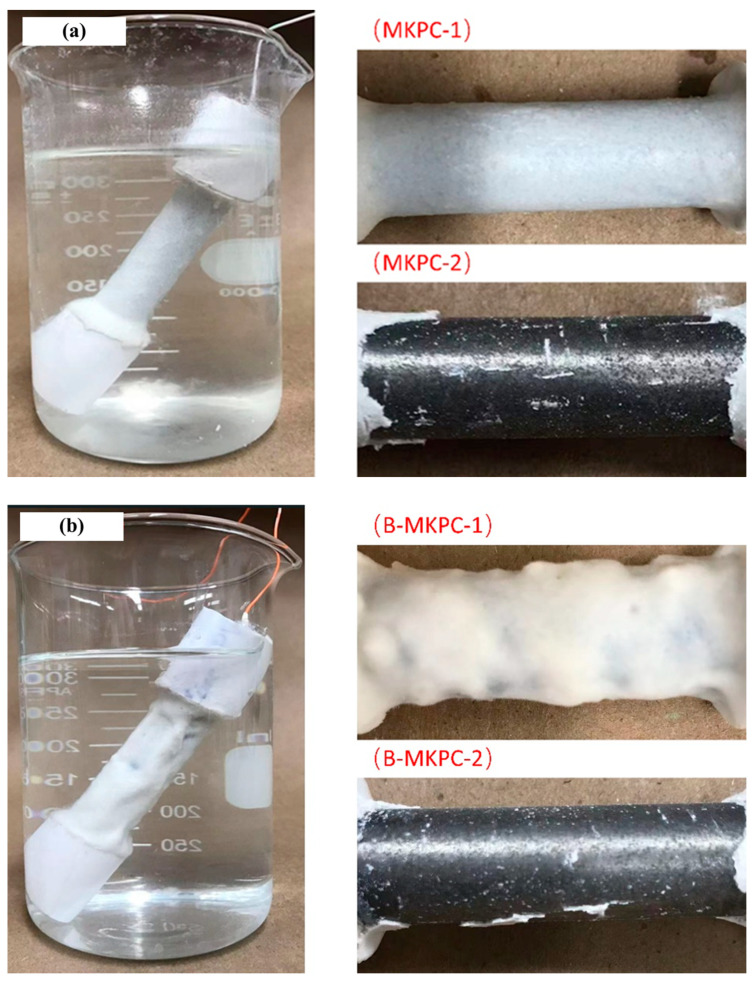
Bar surface conditions (number 1: samples with coating; number 2: samples with peeling off the coating) of samples after 10,080 h EC test for (**a**) MKPC and (**b**) B-MKPC samples.

Figure 14 shows the testing situation, the samples before removing the coating, and the tested plain round bars with the coating layer peeled at the conclusion of the EC test after 10,080 h (420 days). No significant expansive deformation or cracks were noticed for the coating layers. Also, no rust spots were apparent on the outer surface of the MKPC and B-MKPC samples. This observation verified the good corrosion resistance performance of MKPC paste coating as indicated by the polarization resistance and corrosion current density results. Meanwhile, the coating layer of the MKPC sample was relatively smooth with uniform thickness, while the outer surface of the B-MKPC sample was rough and uneven due to the presence of boric acid, which reduces the flowability of fresh paste. A more even and smoother surface may have a positive effect on the corrosion resistance. Thus, modifying the fluidity of fresh MKPC paste may increase its anti-corrosion ability. In addition, no significant rust was observed on the surface of substrate bars. Most of the steel surfaces for both samples appeared to have metallic luster, indicating that the bars were still under protection by MKPC paste coating. This result is consistent with a previous study [14] in which the vast majority of the steel bar surface for a magnesium ammonium phosphate cement remains passivated after immersion in 3.5 wt.% NaCl solution for 450 days.

**Figure 15:**
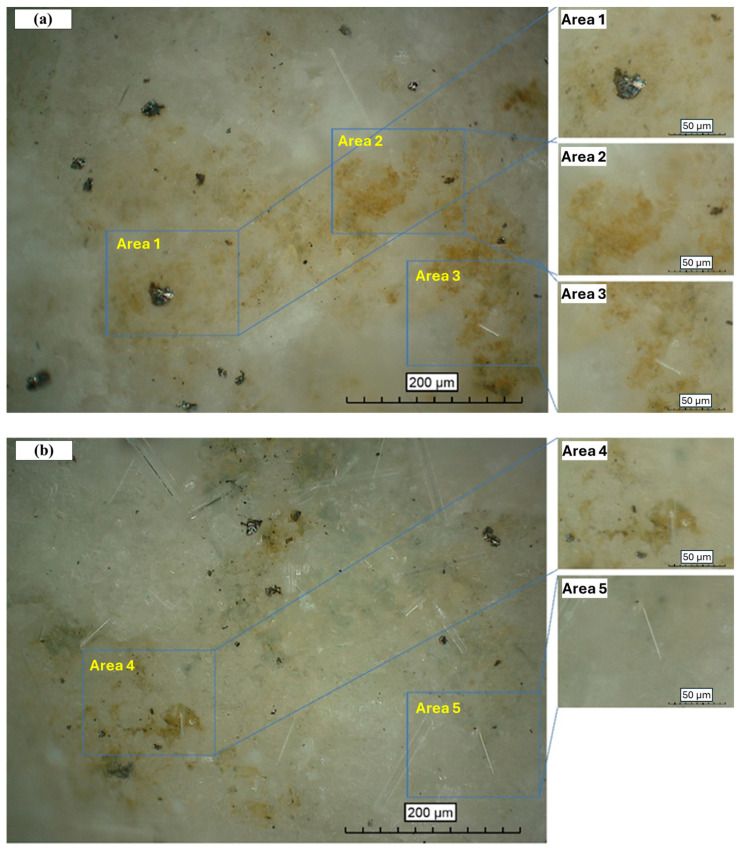
Inner coating layer conditions of samples after 10,080 h EC test for (**a**) MKPC and (**b**) B-MKPC samples.

Figure 15 shows the inner surface of MKPC and B-MKPC paste coating where the coating layer was in contact with the substrate steel after 10,080 h (420 days) of the EC test. At a magnification of 500, some corrosion spots were observed. The inner surface of MKPC revealed some black spots, likely from pitting corrosion. Its formation was likely a result of aggressive ions transferring through connected channels and pores in the coating layer, initiating more active corrosion sites. The yellow part represented the uniform corrosion state, which covered a large part of the observed surface. Compared to the pitting corrosion, which can result in a severe compromise in reinforcement strength [35,36,37], uniform corrosion is relatively less harmful to the structure. Meanwhile, due to the formation of the passive layer between the MKPC paste and substrate steel, most of the inner coating layer remained the original white color, which is consistent with the previous section. The coating layer of the B-MKPC samples was similar to that of the MKPC samples. However, some needle-like substances were found on the coating layer, which is presumably an efflorescence formed due to the low M/P ratio. The nature of efflorescence has a very negative influence on the strength and volume stability of materials and has not been well investigated yet [38]. It is obvious from Figure 15 that the needle-like substances appeared more frequently when the MKPC paste was blended with boric acid. The anti-corrosion performance may be improved by preventing the formation of efflorescence. Judged by the corrosion degree and anti-corrosion performance from the EC test, the presence of more efflorescence was likely to produce more pores, leading to a drop in corrosion resistance.

## 4. Conclusions

This study conducted experimental research on the anti-corrosion performance of MKPC paste coatings without and with boric acid, with a focus on the influences of boric acid. The coating layers were systematically characterized using IC, TG/DTA, XRD and MIP tests. The changes in OCP value, EIS spectrum and polarization resistance were employed to evaluate the corrosion resistance of MKPC and B-MKPC samples. Furthermore, the PDP and visual inspection provided further analysis of the corrosion degree and rate after 10,080 h (420 days) of the EC test.

Even though the presence of boric acid significantly delayed the hydration rate and reduced the heat release within a short time, the chemical compositions of hardened pastes after 7 days of curing were identical. Furthermore, the addition of boric acid increased the amount of hydration products as well as the total porosity from 3.65% to 4.53%. This is likely attributed to the appearance of more efflorescence as a result of the formation of intermediate products.

Judged from the EC test results, the presence of boric acid decreased the anti-corrosion performance and had a negative effect on the polarization resistance of the MKPC coating sample since the initial stage of the EC test. Even though the coating resistance increased during the entire 10,080 h test, a huge drop in polarization resistance was detected. This means that the relatively thick coating layer can protect the substrate reinforcement to some degree, but the increase in porosity during the early stage deteriorates the anti-corrosion performance.

The presence of boric acid in MKPC displays prominent advantages to surmount the insufficient setting time and to modify the heat-releasing process. However, adding boric acid into MKPC remarkably reduced the passivation state time and cannot control the current density below the threshold from the beginning of the test. In summary, in this testing situation, the anti-corrosion performance was deteriorated by mixing boric acid into the MKPC paste coating.

## Figures and Tables

**Table 1:** Chemical composition of the DBM, KDP, and metakaolin (wt.%).

Compound	MgO	SiO_2_	CaO	Al_2_O_3_	Na_2_O	K_2_O	Fe_2_O_3_	SO_3_	P_2_O_5_	TiO_2_	Other
Magnesia	91.90	4.70	1.16	1.07	0.65	-	0.23	0.13	0.11	-	0.05
KH_2_PO_4_	-	2.51	0.17	0.63	0.84	41.98	-	0.21	53.61	-	0.06
Metakaolin	0.27	52.93	-	44.31	-	0.15	0.52	0.16	-	1.47	0.19

**Table 2:** Parameters extracted from potentiodynamic polarization curves for MKPC and B-MKPC specimens.

Time (hours)	OCP (V_SCE_)		*E*_corr_ (mV_SCE_)		*I*_corr_ (µA/cm^2^)		Corrosion Rate (mm/year)	
	MKPC	B-MKPC	MKPC	B-MKPC	MKPC	B-MKPC	MKPC	B-MKPC
840	−0.854	−0.878	−968.427	−979.896	0.419	0.854	4.860 × 10^−3^	9.904 × 10^−3^
5376	−0.828	−0.858	−983.061	−977.837	0.478	1.364	5.550 × 10^−3^	15.819 × 10^−3^
10,080	−0.808	−0.833	−946.563	−942.542	0.618	1.488	7.175 × 10^−3^	17.267 × 10^−3^

## Data Availability

The original contributions presented in the study are included in the article, further inquiries can be directed to the corresponding authors.
